# Looseness Monitoring of Bolted Spherical Joint Connection Using Electro-Mechanical Impedance Technique and BP Neural Networks

**DOI:** 10.3390/s19081906

**Published:** 2019-04-22

**Authors:** Jing Xu, Jinhui Dong, Hongnan Li, Chunwei Zhang, Siu Chun Ho

**Affiliations:** 1 School of Civil Engineering, Qingdao University of Technology, Qingdao 266033, China; jingxu180@qut.edu.cn (J.X.); dongjinjs@hotmail.com (J.D.); 2 State Key Laboratory of Coastal and Offshore Engineering, Dalian University of Technology, Dalian 116024, China; hnli@dlut.edu.cn; 3 Department of Mechanical Engineering, University of Houston, Houston, TX 77004, USA

**Keywords:** structural health monitoring (SHM), space grid structures, electro-mechanical impedance (EMI), back propagation neural networks (BPNNs), bolted spherical joint (BSJ), Bolt looseness damage

## Abstract

The bolted spherical joint (BSJ) has wide applications in various space grid structures. The bar and the bolted sphere are connected by the high-strength bolt inside the joint. High-strength bolt is invisible outside the joint, which causes the difficulty in monitoring the bolt looseness. Moreover, the bolt looseness leads to the reduction of the local stiffness and bearing capacity for the structure. In this regard, this study used the electro-mechanical impedance (EMI) technique and back propagation neural networks (BPNNs) to monitor the bolt looseness inside the BSJ. Therefore, a space grid specimen having bolted spherical joints and tubular bars was considered for experimental evaluation. Different torques levels were applied on the sleeve to represent different looseness degrees of joint connection. As the torque levels increased, the looseness degrees of joint connection increased correspondingly. The lead zirconate titanate (PZT) patch was used and integrated with the tubular bar due to its strong piezoelectric effect. The root-mean-square deviation (RMSD) of the conductance signatures for the PZT patch were used as the looseness-monitoring indexes. Taking RMSD values of sub-frequency bands and the looseness degrees as inputs and outputs respectively, the BPNNs were trained and tested in twenty repeated experiments. The experimental results show that the formation of the bolt looseness can be detected according to the changes of looseness-monitoring indexes, and the degree of bolt looseness by the trained BPNNs. Overall, this research demonstrates that the proposed structural health monitoring (SHM) technique is feasible for monitoring the looseness of bolted spherical connection in space grid structures.

## 1. Introduction

**Figure 1:**
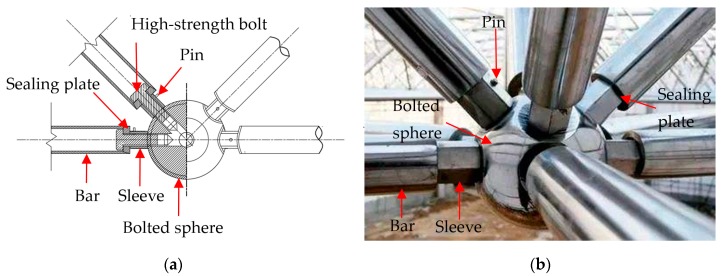
(**a**) Diagram of bolted spherical joint parts; (**b**) Photo of bolted spherical joint of the roof at Qingdao airport terminal-A.

Bolted spherical joints (BSJs) have been widely used in different kinds of space grid structures such as theaters, exhibition centers, airport terminals, highway toll stations, and even structures in space [1,2,3]. BSJs have the advantages of high installation accuracy, good bearing capacity, and flexible connection directions [4,5]. Figure 1a shows a diagram of bolted spherical joint parts. With screwing the sleeve, the high-strength bolt is driven to connect the bar with the bolted sphere successfully. Figure 1b shows the practical application of a bolted spherical joint of the roof at Qingdao airport terminal-A. From Figure 1b, the high-strength bolt is invisible outside the joint, which leads to the difficulty in monitoring the internal bolt looseness. Moreover, the bolt looseness causes the reduction of the local stiffness and bearing capacity for the structure [6,7,8,9]. With the passage of time, the failure of the joint connection, which is often a brittle failure without any indication, is likely to occur suddenly, consequently, leading to serious failure of the structure. Therefore, it is necessary to develop a real-time and effective monitoring technique for the looseness of the bolted spherical joint connection.

At present, structural health monitoring technologies, which can deliver early warning signs using advanced algorithms based on the real time data obtained from integrated transducers, have been widely used in many fields, such as pipeline structures [10,11,12,13], underground engineering [14,15,16], reinforced concrete structures [17,18,19,20,21], and steel-concrete composite structures [22,23,24,25]. Based on the wavelet packet energy analysis method, Xu et al. proposed a very sensitive damage index to monitor the slipping damage between the concrete core and the steel tube in the steel-concrete composite structure [26]. Jiang et al. used an active sensing approach to monitor the rebar corrosion in a prestressed concrete structure, which has great potentials of engineering application in providing the early warning of the initial damage of structures [27]. By using smart aggregates (SAs) and polyvinylidene fluoride (PVDF) thin-film sensors, Qi et al. investigated polyvinyl alcohol (PVA)-reinforced engineering cementitious composite (PVA-ECC) under low-velocity impact loading, which exhibits improved impact resistance [28]. For BSJs in space grid structures, since the high-strength bolt is located inside the joint, the bolt looseness is difficult to detect. The German Mero system opened the holes in the bar near the joint to observe the connection status of the bolted spherical joint. However, this method can not only destroy the integrity of bolted spherical joint connections, but also reduce the structural stiffness [29]. Japanese engineers inserted an endoscope into the nut hole to check the bolt location to detect the joint connection status in the Dalian TV Tower engineering, which achieved good results [30]. However, the cost of using this method is high and it requires skillful operators. Therefore, it is not suitable for real time monitoring. In this research, a novel method to monitor the looseness of BSJs in real time was developed based on the electro-mechanical impedance (EMI) technique and back propagation neural networks (BPNNs) using integrated piezoceramic transducers.

By analyzing the difference of mechanical impedance signatures before and after structural damage, the EMI-based structural health monitoring (SHM) technique can be used to monitor the structural damage [31,32,33,34]. In the early 1990s, the Center for Intelligent Materials Systems and Structures at the Virginia Tech University pioneered the application of piezoelectric impedance technique in structural health monitoring [35]. In recent years, Li et al. employed the impedance method to monitor the debonding status between the concrete and the fiber-reinforced rebar in reinforced concrete (RC) structures, and the presented results demonstrating that the method is feasible for monitoring the debonding status [36]. Shi et al. successfully employed the EMI technique to monitor the grout compactness of concrete-filled fiber-reinforced polymer tubes [37]. Liang et al. proposed the EMI-based SHM to study the bond-slip phenomenon in steel-concrete composite structures and verified the applicability of the method [38]. Yang et al. presented a study on monitoring damage propagation in aluminum plates using the EMI technique and employed a semi-analytical EMI model to predict the lead zirconate titanate (PZT) admittance signatures. Both experimental and predicted signatures were analyzed qualitatively and quantitatively using a statistical method. The results demonstrated the capability of the EMI method for monitoring damage propagation [39]. Annamdas et al. proposed faster measurements by reducing unnecessary resonant structural peaks and focusing on rapid monitoring using wired PZT and wireless localized surface plasmon (LSP). They presented integrated and complementary nature of these techniques, which can be processed rapidly for key infrastructures with great effectiveness. This integration can result in both continuous and delayed SHM techniques based on time, frequency, or both domains [40]. Yang et al. adopted the combination of smart fiber Bragg grating (FBG) and PZT sensors to provide comprehensive health monitoring of rocks, covering load history monitoring/retrieval as well as damage assessment. The experimental study demonstrated superior performance of these smart FBG and PZT impedance sensors [41]. Other mathematical and numerical simulations of PZT-EMI application have been reported [42,43,44]. The EMI technique using piezoceramic transducers is a real-time monitoring method that has the characteristics of easy installation, reliable operation, and high sensitivity to small damages [45,46].

However, changes in temperature and humidity may change the experimental results [47,48]. Haider et al. presented an experimental and analytical study of irreversible change in piezoelectric wafer active sensor (PWAS) electromechanical (E/M) impedance and admittance signature under high temperature exposure [49]. Huynh et al. presented the quantification of temperature effect on impedance monitoring via a PZT interface for prestressed tendon-anchorage and proposed the effective frequency shift-based algorithm to filter temperature effects on impedance monitoring [50]. Huynh et al. proposed a principal component analysis (PCA)-based algorithm to filter out temperature effects on EMI monitoring of prestressed tendon anchorages [51]. Moreover, the bolt loosening is influenced by many factors such as service loads, material aging, and installation errors. These effects on bolted spherical joint looseness monitoring will cause the complex nonlinear relationship between the looseness index and the looseness statuses. In this paper, the trained BPNNs were used to map the nonlinear relationship for its good ability of nonlinear approximation, self-learning, generalization, fault tolerance, and robustness [52,53,54]. Therefore, these effects may be compensated by additional training of the BPNNs.

The EMI and BPNNs-based SHM techniques have not been applied to the monitoring of the looseness of BSJs in a space grid structure. Based on EMI technique and BPNNs, a novel method to monitor the looseness of BSJs in space structures is proposed in this paper. According to the changes of looseness-monitoring indexes proposed for the EMI technique, the occurrence of bolt looseness can be detected. Based on the trained BPNNs, the degree of bolt looseness can be determined.

## 2. Detection Principle

### 2.1. Electro-Mechanical Impedance

**Figure 2:**
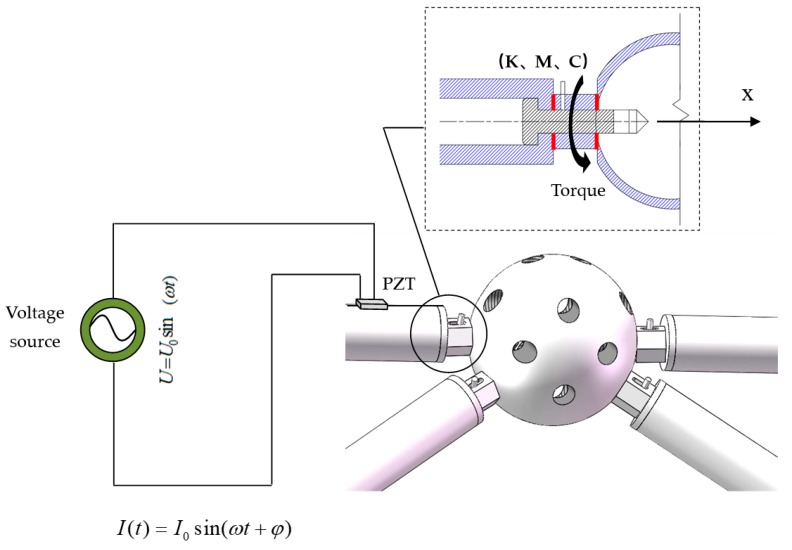
The schematic diagram of electro-mechanical impedance EMI technique by applying the lead zirconate titanate PZT patch.

The EMI method detects the damage in the structure by analyzing the difference of mechanical impedance signatures before and after the structural damage [55,56,57]. This method works on the basis of the direct and inverse piezoelectric effect of the piezoelectric transducers [58]. This method is especially sensitive to the localized small damage in the structure [59,60]. Among all piezoelectric materials, PZT has a strong piezoelectric effect and wide bandwidth [61,62], it is also often used to generate and sense stress waves [63,64,65]. Considering their advantages, PZT transducers is often used in EMI methods [66,67,68]. In addition, PZT materials are available in different shapes, sizes, and patches. Such materials are small and sensitive, often used in the structures for damage detection [69,70]. The electro-mechanical coupling effect in EMI works on the principle that the mechanical energy and electrical energy can be converted into each other between the host structure and PZT patches. Figure 2 shows the schematic diagram of the EMI technique by applying the PZT patch. The relationship between the admittance Y(ω) (reciprocal of impedance) of the PZT patch and the mechanical impedance $Z_{a}(\omega )$, $Z_{s}(\omega )$ of the PZT patch and the host structure can be expressed as follows [35]:

(1) $$Y(\omega )=j\omega \frac{wl}{h}\left\{{\bar{\varepsilon }}_{33}^{T}-d_{31}^{2}{\bar{Y}}^{E}+\left(\frac{Z_{a}(\omega )}{Z_{s}(\omega )+Z_{a}(\omega )}\right)\;d_{31}^{2}{\bar{Y}}^{E}\left(\frac{\tan kl}{kl}\right)\right\}$$

where ω represents the angular frequency under driving voltage, Y(ω) is the electrical admittance (reciprocal of impedance) of PZT patches at ω angular frequency, and $Z_{a}(\omega )$, $Z_{s}(\omega )$ represent the mechanical impedance of PZT patches and host structure at ω angular frequency respectively. w,l,h represent the width, length, and thickness of PZT patches, respectively. δ is the dielectric loss factor of the PZT patch. ${\bar{\varepsilon }}_{33}^{T}=\varepsilon_{33}^{T}(1-\delta )$ is the complex electric permittivity when the stress is equal to zero. η represents the mechanical loss factor of the PZT patch. ${\bar{Y}}^{E}=Y^{E}(1+\eta )$ is the complex young’s modulus of PZT patch when the electric field is equal to zero. $d_{31}$ is the piezoelectric constant of the PZT patch and $K=\omega \sqrt{\rho /{\bar{Y}}^{E}}$ is the wave number, where ρ is the mass density of the PZT patch.

It can be seen in Equation (1) that, when the material properties of PZT patches remain unchanged, the mechanical impedance of the structure uniquely determines the electrical impedance of the PZT patch. Therefore, the electrical impedance change of the PZT patch provides the mechanical impedance change of the structure.

### 2.2. Monitoring the Looseness of Bolted Spherical Joints Based on Electro-Mechanical Impedance

According to a large number of studies [19], the mechanical impedance of host structure can finally be represented by the ratio between the force and the final velocity. The mechanical impedance of host structure can be expressed as follows:

(2) $$Z_{s}(\omega )=-\frac{F}{\dot{x}}=c+\frac{m\omega^{2}-K_{m}}{\omega }i$$

where F is the force applied, $\dot{x}$ is the final velocity, ω is the angular frequency, c is the damping coefficient, m is the mass, $K_{m}$ is the static stiffness, and i is the imaginary number.

When the bolt looseness occurs, the local stiffness of structure in the position of bolted spherical joint gets weakened [71]. According to Equation (2), it will lead to the changes of structural mechanical impedance. Since the electrical impedance change of the PZT patch can reflect the mechanical impedance change of the structure, the bolt looseness inside the joint can be monitored by comparing the electrical impedance change of the PZT patch before and after the bolt loosening. It can help in solving the problems of bolt looseness. Moreover, it can also help in avoiding the inconvenience of monitoring the host structure directly.

### 2.3. Damage Identification Index

Only quantitative analysis of change in structural impedance characteristics can accurately measure the extent of structural damage. Therefore, root-mean-square deviation (RMSD) and mean-absolute-percentage deviation (MAPD) were used to quantitatively analyze the changes of PZT impedance signatures. The mathematical expression represented by the real admittance part Re(Y) received from PZT can be expressed as follows:

(3) RMSD(%)=∑i=1N [Re(Yi,1)−Re(Yi,0)]2∑i=1N [Re(Yi,0)]2

(4) MAPD(%)=1N∑i=1N |[Re(Yi,1)−Re(Yi,0)]Re(Yi,0)|

where N represents the total number of impedance spectrum sampling points, Re(Yi,0) represents the admittance value under the healthy status, and Re(Yi,1) represents the admittance value under the actual status. Re(Yi,1)−Re(Yi,0) is the difference between the healthy status and the actual status.

For looseness monitoring of BSJs, it should be noted that the looser the bolt, the higher the damage. As a result, Re(Yi,1)−Re(Yi,0), which shows the difference between the healthy status and the actual status, will increase and the looseness index, RMSD, and MAPD will become significantly larger. Therefore, the increase in the bolt looseness will lead to the increase in RMSD and MAPD.

### 2.4. Bolted Spherical Joint Looseness Monitoring Using BPNNs

BPNNs has the powerful nonlinear mapping capacity and has wide applications [72,73,74]. The three layers of BPNNs are an input layer, a hidden layer, and an output layer. BPNNs are trained with a momentum, a maximum number of iterations, adaptive learning rate, and a back-propagation algorithm. Essentially, the learning rate is an important factor for the convergence rate of the networks and for the convergence to a global minimum of the objective function. The performance of BPNNs depends on the training samples’ number and multiplicity in the database. In this paper, BPNNs is used to map the nonlinear relationship between conductance signatures and the degrees of bolt looseness. Using the trained BPNNs, the looseness degree of BSJs can be determined.

## 3. Experimental Setup

### 3.1. Specimen

**Figure 3:**
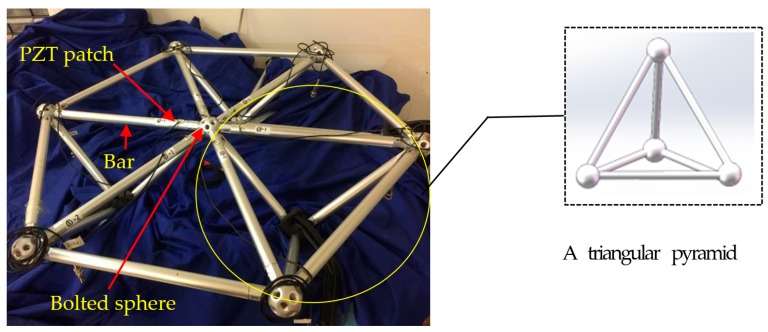
Photo of triangle-on-triangle offset grid specimen.

To verify the feasibility of the EMI and BPNNs-based SHM technique for looseness monitoring of bolted spherical joint connection, the experiment was conducted on a space grid specimen. Figure 3 depicts the geometry details of experimental specimen. The steel material used was Q345. The specimen was composed of three triangles on triangle offset units. The dimensions of bars were Φ22 mm by 6 mm. The dimensions of bolted spheres were Φ46 mm by 10 mm. In order to simulate the fixed support, clamps were used to fix the specimen on the experimental table. The thick sponges were used to separate the specimen from the clamps in order to improve the experiment’s accuracy.

### 3.2. Bolted Sphere Experiment Setup

**Figure 4:**
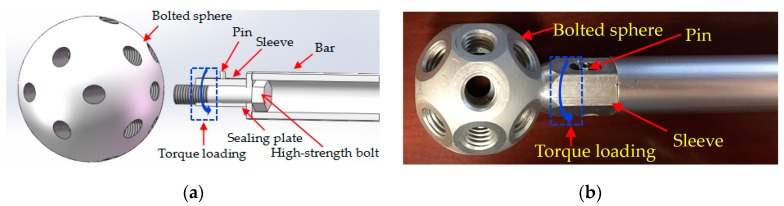
(**a**) Installation schematic representation of bolted spherical joint (**b**) Photo of bolted spherical joint connection.

The schematic representation of the installation of bolted spherical joint has been shown in Figure 4. By screwing the sleeve, the high-strength bolt with the bar becomes closer to the bolted sphere as it is continuously inside the joint until the installation of the bolted spherical joint is completed successfully. During the installation of the bolted spherical joint, the process of the sleeve screwing was divided into two stages: The initial stage and the final stage. In the initial stage, the sleeve was screwed by hand. In the final stage, the sleeve was screwed by the torque wrench. For the final stage, the bolt looseness was small and therefore it was very difficult to detect the small bolt looseness outside the joint. The accumulation of small looseness will lead to the connection failure and will affect the structural durability.

The EMI technique has the characteristics of higher working frequency and better sensitivity to small damage. In this paper, it was used to monitor and identify the small bolt looseness occurring in the final screwing stage. Different torque levels represent different looseness statuses. At first, the joint connection was restored to the healthy status. Then, by Craftsman’s Model 32,999 Torque Wrench with different torque levels was applied on the sleeve to loosen the bolt. Different looseness degrees with an increasing torque level from 3.39 N·m (2.5 lb·ft) to 23.75 N·m (17.5 lb·ft) with increments of 2.5 lb·ft were considered in this research. Under the increase of the torque level, the bolt looseness increased accordingly. The experiment was performed in controlled laboratory conditions at the temperature of 25 °C and the temperature was stable during the measurement.

### 3.3. EMI Monitoring

**Figure 5:**
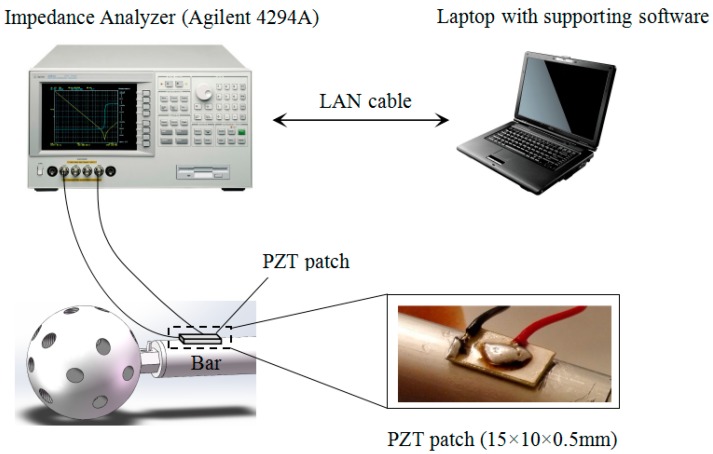
The schematic of bolt looseness monitoring inside the spherical joint based on EMI technique.

**Figure 6:**
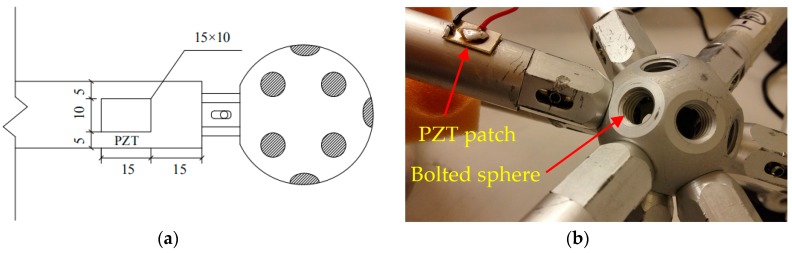
(**a**) Detailed dimension of the location of PZT patch (unit: mm);(**b**) Photo of the location of PZT patch.

**Figure 7:**
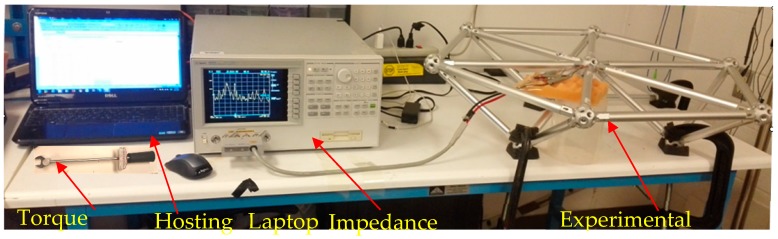
Experimental setup.

Figure 5 shows the EMI measurement system. It was composed of an impedance analyzer (Agilent 4294A, Keysight Technologies, Santa Rosa, CA, USA), a laptop with supporting software and a PZT patch. The PZT patch, which has a size of 15 mm by 10 mm by 0.5 mm, was bonded very close to the bolted sphere on the surface of the bar using epoxy resin. At first, we sanded the bonding area on the surface of the bar in order to improve the bonding. Then, the PZT patch was bonded very close to the bolted sphere on the surface of the bar using epoxy resin. At last, we used epoxy resin to reinforce the entire bonding area from the outside. The detailed dimensions of the location of PZT patch is shown in Figure 6. The high-frequency AC electric field (i.e., converse piezoelectric effect) was applied to the PZT patch by the impedance analyzer and the mechanical vibration was generated on PZT patch. Accordingly, the connected bar also vibrates. Conversely, the vibration of bar causes the PZT patch to produce the electrical responses (i.e., direct piezoelectric effect), which was represented by the change of electrical impedance for the PZT patch. The electrical impedance signatures of the PZT patch were measured by the impedance analyzer and were stored in the computer. By comparing the electrical impedance signatures of PZT patch before and after damage, the bolt looseness was monitored. Figure 7 shows the experimental setup.

**Figure 8:**
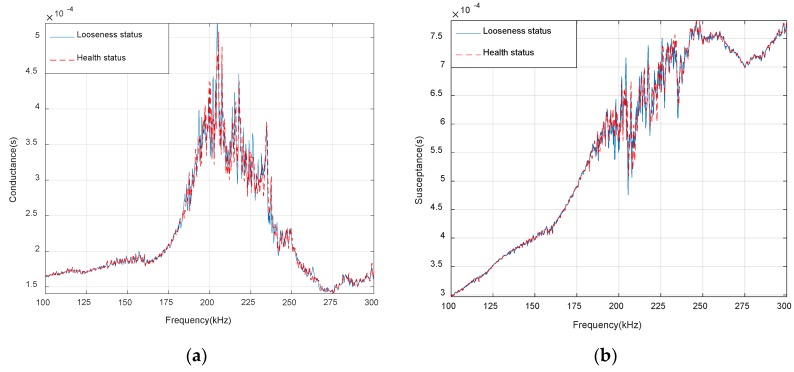
(**a**) Conductance signatures of PZT patch between healthy status and looseness status (100–300 kHz); (**b**) Susceptance signatures of PZT patch between healthy status and looseness status (100–300 kHz).

For the EMI–based SHM technique, the accuracy of the monitoring results is closely related to the sensitive frequency range and the measured signature. The resonant frequencies of the patch were 200–250 kHz. In order to select a suitable frequency range, the PZT patch on the specimen was scanned over a wide frequency range of 100–300 kHz. The conductance and susceptance signatures, which are the real part and imaginary of admittance, of the PZT patch between healthy and looseness status are shown in Figure 8a,b. There are 801 sampling points in the signature spectra. The admittance is the reciprocal of impedance. Figure 8a shows that the conductance signatures have multiple sharp peaks in the frequency range between 200–220 kHz. Compared with Figure 8a,b, it can be seen that the conductance is more sensitive than the susceptance. This is due to the fact that the susceptance is dominated by the capacitive response of the sensor and thus is less sensitive to the changes in the mechanical properties of the structure [75]. Therefore, the frequency range of 200–220 kHz was selected as the sensitive frequency range and the conductance was selected as the measured signature in the following study.

## 4. Experimental Results and Analysis

### 4.1. Looseness-Monitoring Index

**Figure 9:**
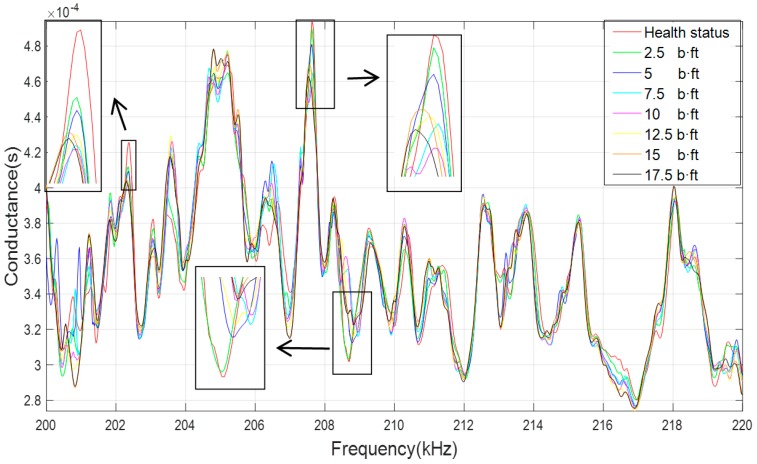
Conductance signatures at different torque level (200–220 kHz).

According to the experiment procedure described in Section 3.2, different looseness degrees with an increasing torque level from 3.39 N·m (2.5 lb·ft) to 23.75 N·m (17.5 lb·ft) with increments of 2.5 lb·ft were considered. Figure 9 shows the conductance signatures over the sensitive frequency range of 200–220 kHz for different looseness degrees. It can be seen that each torque level is separable from others with the curves.

**Figure 10:**
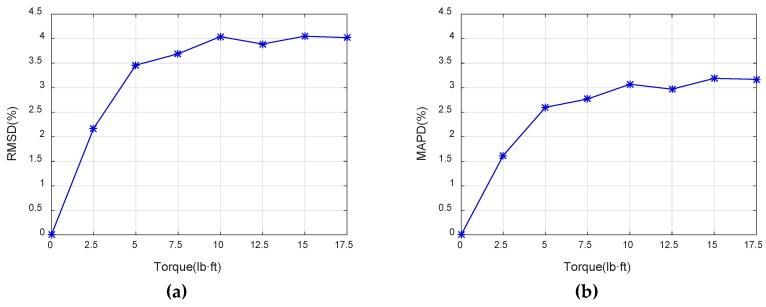
(**a**) The root-mean-square deviation (RMSD) of conductance signatures at different torque levels; (**b**) The mean-absolute-percentage deviation (MAPD) of conductance signatures at different torque levels.

The bolt looseness was quantified according to the monitoring indexes (RMSD and MAPD) in this paper. Taking the healthy state as the baseline, the RMSD and MAPD of conductance signatures over 200–220 kHz were calculated using Equations (3) and (4) for each torque level, as shown in Figure 10. As the torque level increases, the conductance-based looseness indexes (RMSD and MAPD) increases. This is due to the fact that greater torque leads to the looser joint connection. Based on the EMI method, the looseness indexes (RMSD and MAPD) can effectively quantify the loosening degree of bolted spherical joint connection. Figure 10 also shows that the RMSD and MAPD looseness indexes have the same trend, and the RMSD value is larger than the MAPD value for the same torque level. We selected the RMSD as the looseness-monitoring index in this research.

### 4.2. Repeatability Verification

**Figure 11:**
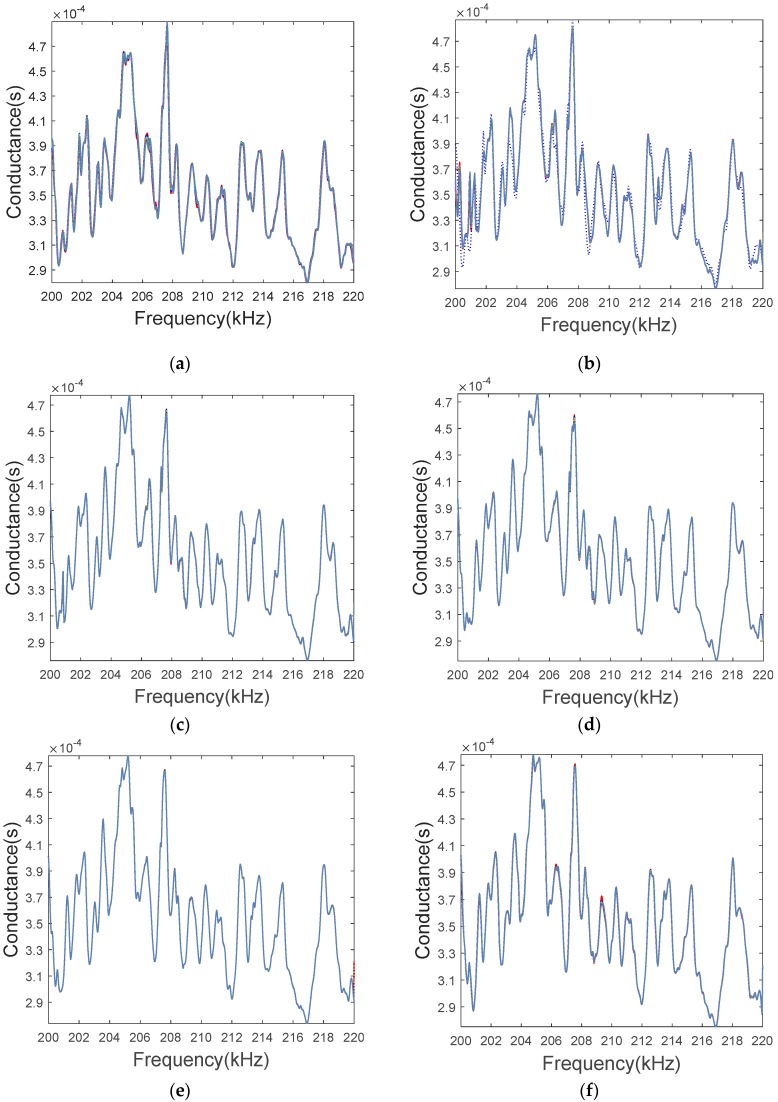

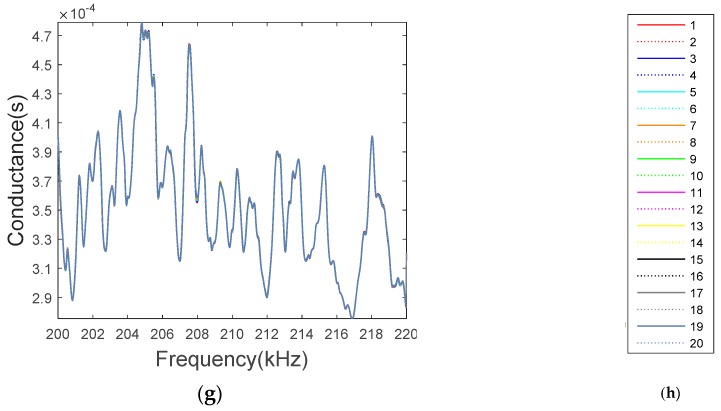
The conductance signatures for twenty repeated experiments at different torque levels. (**a**) 2.5 lb·ft, (**b**) 5.0 lb·ft, (**c**) 7.5 lb·ft, (**d**) 10.0 lb·ft, (**e**) 12.5 lb·ft, (**f**) 15.0 lb·ft, (**g**) 17.5 lb·ft, (**h**) Repetitions (in No.).

**Figure 12:**
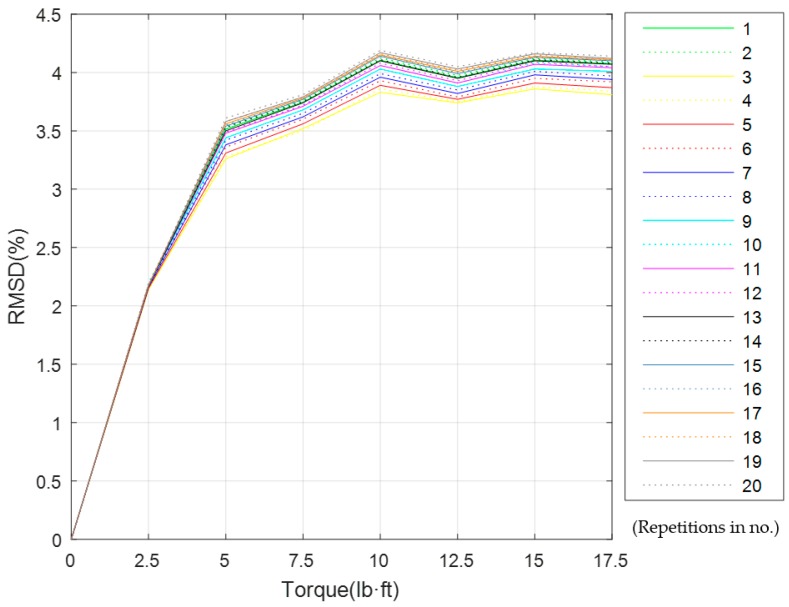
The RMSD looseness index for twenty repeated experiments at different torque levels.

To verify the applications of the EMI method, the experiment was repeated twenty times based on the experimental conditions presented in Section 3.2. Before each experiment, the bolted spherical joint connection was restored to the healthy status. The conductance signatures and RMSD at different torque levels were obtained, as shown in Figure 11 and Figure 12. Figure 11 shows that for the same torque level (i.e., looseness degree), through twenty experiments had very similar conductive responses. Figure 12 shows that with the increase of torque level, the RMSD looseness index increased. However, when the torque becomes higher than 12.5 lb·ft, the sensitivity of RMSD looseness index decreased because of the saturation phenomenon, which has also been reported by other piezoceramic based bolt looseness researches [76,77,78,79]. The saturation is caused by the effective contact area between the two contacting surfaces, which are full of peaks and valleys at a microscale.

### 4.3. Determining the Damage Degree Based on BPNNs

According to Section 4.1 and Section 4.2, the conductance signatures under different torque levels are different and the conductance signatures under the same torque levels are very similar. By using the powerful nonlinear mapping and classification recognition abilities of BPNNs, the different looseness degrees of bolted spherical joint connection were identified in in this research.

**Figure 13:**
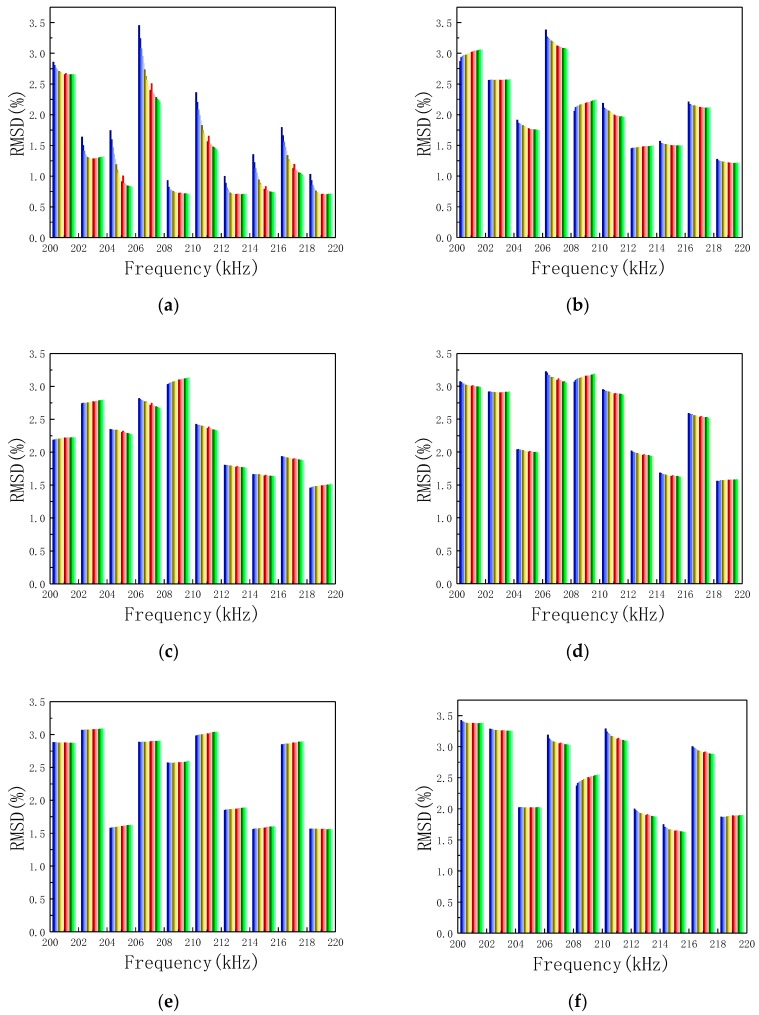

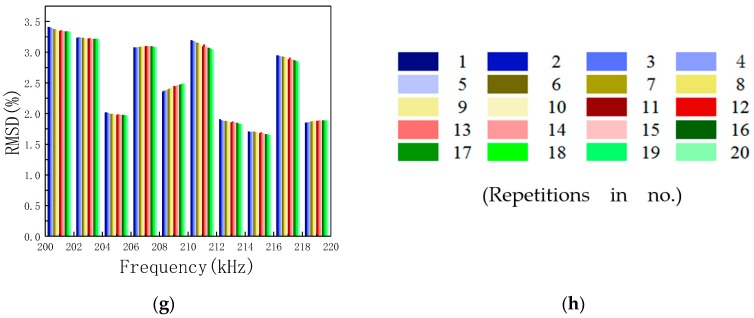
The RMSD values of ten sub-frequency bands at different torque levels for twenty repeated experiments (**a**) 2.5 lb·ft, (**b**) 5.0 lb·ft, (**c**) 7.5 lb·ft, (**d**) 10.0 lb·ft, (**e**) 12.5 lb·ft, (**f**) 15.0 lb·ft, (**g**) 17.5 lb·ft, (**h**) Repetitions (in No.).

**Figure 14:**
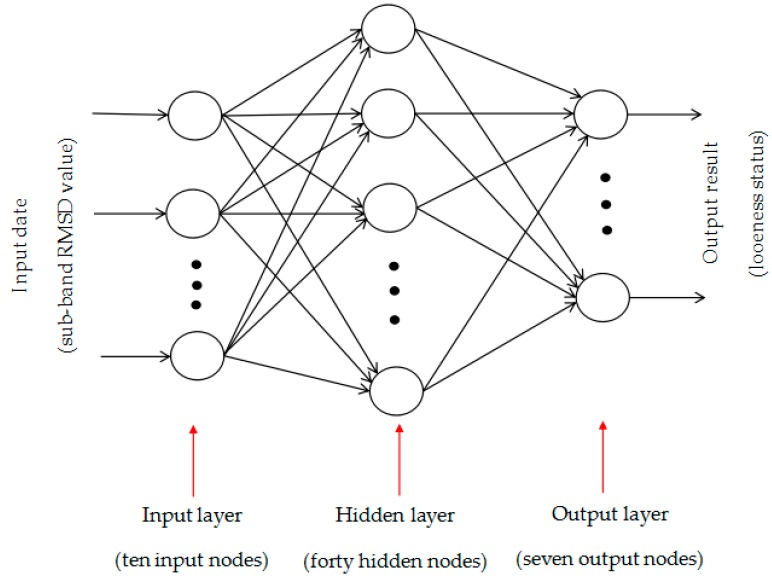
The structure of back propagation (BP) neural networks (10 × 40 × 7).

The sensitive frequency band 200–220 kHz is equally divided into ten sub-frequency bands. Based on the conductance signatures over the sub-frequency bands, ten RMSD values can be calculated using Equation (3). The RMSD values are shown in Figure 13. With the RMSD values over different sub-frequency bands as the inputs and the looseness degrees (torque level), BPNNs were constructed. The structure of back propagation (BP) neural networks is 10 by 40 by 7, as shown in Figure 14.

**Figure 15:**
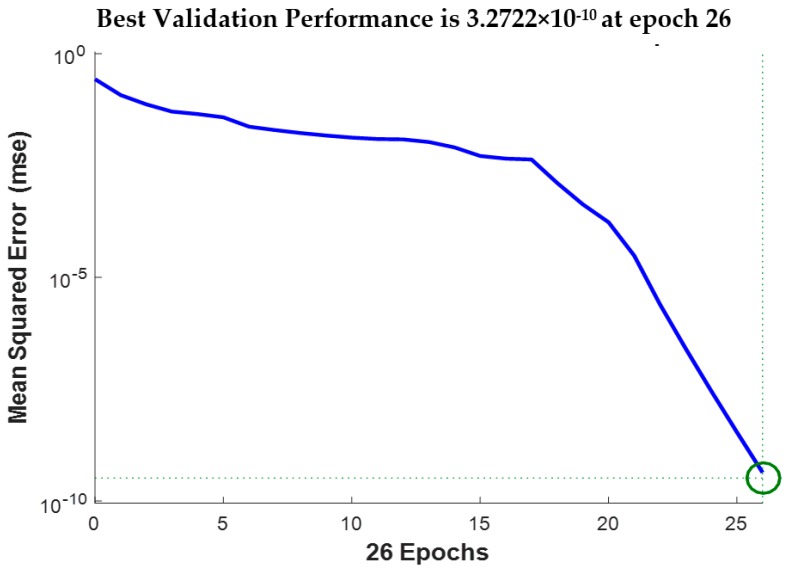
The training convergence curve of BP neural network.

In the twenty repeated experiments, the seventeen experiments were taken as neural networks’ training samples and the remaining three experiments were used as the neural networks’ testing samples. Since there were seven torque levels (i.e., looseness degrees), the total numbers of the training samples and testing samples were 119 and 21, respectively. The training accuracy was 10^−10^. The adaptive learning rate was 0.01. The momentum coefficient was 0.9. The maximum training number of iterations was 26. The back-propagation algorithm was the gradient descent algorithm. Figure 15 shows the networks training process. The training process is stable and convergent. The networks testing results are shown in Table 1. The trained BPNNs model can correctly identify the looseness degrees of all testing samples.

### 4.4. Discussion

In this research, a feasibility study was conducted for the looseness monitoring of bolted spherical joint connection using a PZT-based EMI technique. Experimental results, obtained from twenty repeated test sets, showed that the conductance-based looseness index (RMSD) increases as the looseness degree increases, especially for the initial looseness levels.

The change in the conductance signatures may change with the external conditions, such as loading, operational vibration, noise disturbance, temperature, or humidity. However, these high-frequency-based EMI models are limited to local area. The advantage is that at high frequencies, the measurements are less sensitive to the external condition [80]. However, it was observed that for laboratory specimens where the test structure dimensions are much smaller than the imitated practical structures, the external conditions can also affect the PZT-based signatures [81]. The sensing region of the PZT patch is an important issue of EMI technique. For laboratory specimens (especially those made of metals), the whole structure usually comes under the sensing region of the PZT patch and it can be monitored effectively.

In future work, we will use extensive numerical modeling to identify the sensing region of PZT patch and use additional ultrasonic monitoring systems to enhance monitoring capabilities. The compensation algorithms can counter the changes of external conditions. In this paper, taking the conductance-based looseness index (RMSD) values of sub-frequency bands and the looseness degrees as inputs and outputs respectively, the BPNNs were trained and tested through twenty repeated experiments. The simulated results showed that the degree of bolt looseness can be determined by the trained BPNNs. The changes of external conditions may be compensated by additional training of the BPNNs. In the future work, we will carry out a thorough parametric study to evaluate the amount of compensation through mathematical and numerical simulations.

In addition, as a future work, two approaches will be tried to reduce the adverse effects of saturation, which is seen in Figure 12. The first approach is the use of the nonlinear ultrasonic methods, such as the Vibro-acoustic Modulation (VAM), which is more sensitive to minor damage, i.e., very minor degree of looseness for a bolt connection. The second approach will use an entropy-based method since a loosened bolt connection represents more irregularities at the interface, which means a higher entropy.

## 5. Conclusions

Based on the EMI technique and BPNNs, the looseness of bolted spherical joint connection is monitored. The results show that the method can effectively detect the occurrence and degree of bolt looseness in bolted spherical joint connection. In order to improve the monitoring accuracy, the PZT patch was bonded on the side of the bar near the bolted spherical joint by the epoxy resin. Comparing the electrical responses of PZT patch, the real part of admittance (conductance) is more sensitive than the imaginary part of admittance (susceptance) to monitor the bolt looseness. The RMSD of conductance signatures is used as the looseness-monitoring index under different torque levels. Using the data from twenty repeated experiments, the feasibility and effectiveness of proposed monitoring method is verified. The results show that the looser the connection, the bigger the RMSD value. The RMSD looseness index is more sensitive to initial small damage. The trained BP neural networks are used to map the nonlinear relationship between the conductance signatures and the bolt looseness degrees. When the RMSD of conductance signatures of PZT patch is input into the trained BP neural networks, the looseness degree of bolted spherical joint connection can correctly be determined. This study has a certain reference value for monitoring the looseness of bolted spherical joints in space grid structures.

## Figures and Tables

**Table 1:** The prediction results of trained back propagation neural networks (BPNNs) at different torque levels (the target result of networks in the red boxes).

	Torque Level	2.5 lb·ft	5.0 lb·ft	7.5 lb·ft	10.0 lb·ft	12.5 lb·ft	15.0lb·ft	17.5 lb·ft	Testing Results
Testing Samples									
Looseness degree 1	1	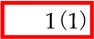	8.53 × 10^−6^(0)	5.05 × 10^−6^(0)	6.29 × 10^−11^(0)	2.43 × 10^−13^(0)	2.78 × 10^−16^(0)	6.20 × 10^−6^(0)	true
	2	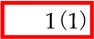	9.03 × 10^−6^(0)	4.95 × 10^−6^(0)	7.13 × 10^−11^(0)	2.82 × 10^−13^(0)	2.78 × 10^−16^(0)	5.51 × 10^−6^(0)	true
	3	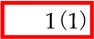	9.09 × 10^−6^(0)	4.88 × 10^−6^(0)	7.06 × 10^−11^(0)	2.66 × 10^−13^(0)	2.78 × 10^−16^(0)	5.92 × 10^−6^(0)	true
Looseness degree 2	1	8.16 × 10^−6^(0)	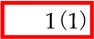	3.06 × 10^−6^(0)	4.17 × 10^−6^(0)	1.04 × 10^−7^(0)	0(0)	4.45 × 10^−6^(0)	true
	2	7.52 × 10^−6^(0)	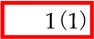	2.98 × 10^−6^(0)	5.11 × 10^−6^(0)	1.33 × 10^−7^(0)	0(0)	3.41 × 10^−6^(0)	true
	3	6.86 × 10^−6^(0)	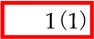	2.86 × 10^−6^(0)	6.55 × 10^−6^(0)	1.77 × 10^−7^(0)	0(0)	2.50 × 10^−6^(0)	true
Looseness degree 3	1	4.16 × 10^−6^(0)	1.00 × 10^−5^(0)	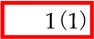	1.22 × 10^−5^(0)	1.75 × 10^−6^(0)	1.37 × 10^−7^(0)	0(0)	true
	2	4.16 × 10^−6^(0)	8.88 × 10^−6^(0)	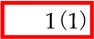	9.83 × 10^−6^(0)	1.90 × 10^−6^(0)	2.51 × 10^−7^(0)	0(0)	true
	3	4.31 × 10^−6^(0)	9.41 × 10^−6^(0)	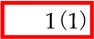	9.90 × 10^−6^(0)	1.61 × 10^−6^(0)	2.21 × 10^−7^(0)	0(0)	true
Looseness degree 4	1	1.92 × 10^−7^(0)	6.00 × 10^−6^(0)	1.96 × 10^−5^(0)	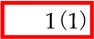	2.46 × 10^−5^(0)	1.31 × 10^−5^(0)	5.22 × 10^−15^(0)	true
	2	1.93 × 10^−7^(0)	6.05 × 10^−6^(0)	2.00 × 10^−5^(0)	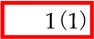	2.46 × 10^−5^(0)	1.32 × 10^−5^(0)	5.11 × 10^−15^(0)	true
	3	1.95 × 10^−7^(0)	5.96 × 10^−6^(0)	2.08 × 10^−5^(0)	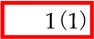	2.42 × 10^−5^(0)	1.33 × 10^−5^(0)	5.00 × 10^−15^(0)	true
Looseness degree 5	1	2.42 × 10^−8^(0)	3.22 × 10^−6^(0)	2.49 × 10^−6^(0)	1.31 × 10^−5^(0)	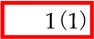	4.52 × 10^−7^(0)	9.73 × 10^−7^(0)	true
	2	2.38 × 10^−8^(0)	2.09 × 10^−6^(0)	4.22 × 10^−6^(0)	2.23 × 10^−5^(0)	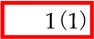	1.77 × 10^−6^(0)	2.31 × 10^−7^(0)	true
	3	2.42 × 10^−8^(0)	2.95 × 10^−6^(0)	2.74 × 10^−6^(0)	1.46 × 10^−5^(0)	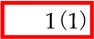	5.80 × 10^−7^(0)	7.42 × 10^−7^(0)	true
Looseness degree 6	1	4.26 × 10^−6^(0)	7.14 × 10^−8^(0)	6.26 × 10^−6^(0)	1.12 × 10^−5^(0)	1.34 × 10^−6^(0)	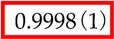	1.42 × 10^−4^(0)	true
	2	4.37 × 10^−6^(0)	6.20 × 10^−8^(0)	7.22 × 10^−6^(0)	1.35 × 10^−5^(0)	1.07 × 10^−6^(0)	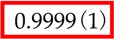	9.85 × 10^−5^(0)	true
	3	4.57 × 10^−6^(0)	6.10 × 10^−8^(0)	6.83 × 10^−6^(0)	1.24 × 10^−5^(0)	9.46 × 10^−7^(0)	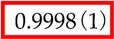	1.30 × 10^−4^(0)	true
Looseness degree 7	1	6.56 × 10^−6^(0)	3.27 × 10^−5^(0)	2.22 × 10^−9^(0)	2.88 × 10^−8^(0)	1.33 × 10^−5^(0)	1.15 × 10^−5^(0)	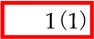	true
	2	6.70 × 10^−6^(0)	2.90 × 10^−5^(0)	2.55 × 10^−9^(0)	4.83 × 10^−8^(0)	8.90 × 10^−6^(0)	1.87 × 10^−5^(0)	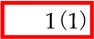	true
	3	6.60 × 10^−6^(0)	2.17 × 10^−5^(0)	3.74 × 10^−9^(0)	6.65 × 10^−8^(0)	7.71 × 10^−6^(0)	4.91 × 10^−5^(0)	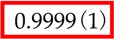	true
